# *N*,*N*,*N*-Tri­butyl­butan-1-aminium (*T*-4)-(cyano-κ*C*)tri­hydro­borate

**DOI:** 10.1107/S1600536813028924

**Published:** 2013-10-26

**Authors:** Thierry Maris

**Affiliations:** a Department of Chemistry, University of Montréal, CP 6128, Succ. Centre-ville, Montréal, Québec, H3C 3J7, Canada

## Abstract

In the crystal structure of the title salt, C_16_H_36_N^+^·CH_3_BN^−^, the tetra-*n*-butyl­ammonium cations and [BH_3_(CN)]^−^ anions are connected *via* weak C—H⋯N inter­actions, forming chains along the *b*-axis direction. The anion is almost linear with an N—C—B angle of 178.7 (2)°. The C—N—C angle values at the core of the tetra-*n*-butyl­ammonium cation range from 105.74 (11) to 111.35 (11)° with an average of 109.49 (11)°, close to the ideal tetra­hedral value.

## Related literature

For the use of the title compound as a reducing agent, see: Hutchins & Kandasamy (1973 ▶). It is also a selective reagent for reductive amination (Hutchins & Markovitz, 1981 ▶) and has been used as a radical mediator for hy­droxy­methyl­ation reactions (Kawamoto *et al.*, 2012 ▶). For the structure of related borohydride salts, see: Jaroń & Grochala (2011 ▶) (tetra­methyl­ammonium) and Jaroń *et al.* (2012 ▶) (tetra-*n*-butyl­ammonium). For the ability of cyano­borohydride anions to form di­hydrogen bonds, see: Custelcean & Jackson (1998 ▶). For the most usual conformations of quaternary ammonium cations, see: Alder *et al.* (1990 ▶).
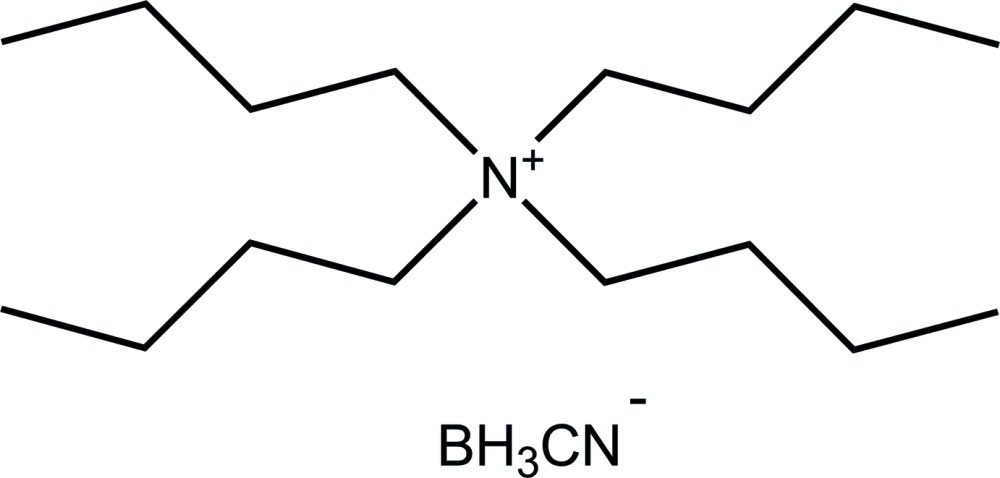


## Experimental

### 

#### Crystal data

C_16_H_36_N^+^·CH_3_BN^−^
*M* *_r_* = 282.31
Monoclinic, 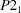

*a* = 7.8312 (5) Å
*b* = 13.9334 (9) Å
*c* = 9.6313 (6) Å
β = 112.269 (2)°
*V* = 972.54 (11) Å^3^
*Z* = 2
Cu *K*α radiation
μ = 0.40 mm^−1^
*T* = 100 K
0.25 × 0.2 × 0.15 mm

#### Data collection

Bruker Microstar X8 diffractometer
Absorption correction: multi-scan (*SADABS*; Sheldrick, 2012 ▶) *T*_min_ = 0.590, *T*_max_ = 0.753
18043 measured reflections
3520 independent reflections
3510 reflections with *I* > 2σ(*I*)
*R*_int_ = 0.041

#### Refinement

*R*[*F* ^2^ > 2σ(*F* ^2^)] = 0.035
*wR*(*F* ^2^) = 0.092
*S* = 1.03
3520 reflections
186 parameters
1 restraint
H-atom parameters constrained
Δρ_max_ = 0.17 e Å^−3^
Δρ_min_ = −0.16 e Å^−3^
Absolute structure: Flack parameter determined using 1596 quotients [(I^+^)−(I^−^)]/[(I^+^)+(I^−^)] (Parsons & Flack, 2004 ▶)
Absolute structure parameter: 0.14 (12)

### 

Data collection: *APEX2* (Bruker, 2013 ▶); cell refinement: *SAINT* (Bruker, 2013 ▶); data reduction: *SAINT*; program(s) used to solve structure: *SHELXS2013* (Sheldrick, 2008 ▶); program(s) used to refine structure: *SHELXL2013* (Sheldrick, 2008 ▶); molecular graphics: *OLEX2* (Dolomanov *et al.*, 2009 ▶); software used to prepare material for publication: *OLEX2* and *publCIF* (Westrip, 2010 ▶).

## Supplementary Material

Crystal structure: contains datablock(s) I, global. DOI: 10.1107/S1600536813028924/sj5361sup1.cif

Structure factors: contains datablock(s) I. DOI: 10.1107/S1600536813028924/sj5361Isup2.hkl

Supplementary material file. DOI: 10.1107/S1600536813028924/sj5361Isup3.cdx

Additional supplementary materials:  crystallographic information; 3D view; checkCIF report

## Figures and Tables

**Table 1:** Hydrogen-bond geometry (Å, °)

*D*—H⋯*A*	*D*—H	H⋯*A*	*D*⋯*A*	*D*—H⋯*A*
C1—H1*B*⋯N2^i^	0.97	2.58	3.515 (2)	162
C2—H2*B*⋯N2	0.97	2.58	3.523 (2)	165
C13—H13*B*⋯N2	0.97	2.59	3.474 (2)	152

Symmetry code: (i) 
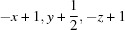
.

**Fig. 1.**
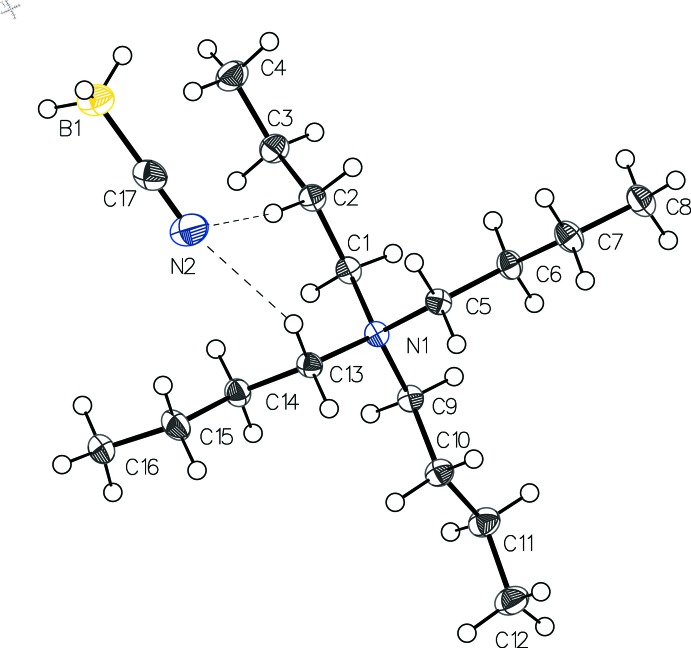
The molecular structure of the title compound, with atomic numbering scheme and 50% probability displacement ellipsoids for non-hydrogen atoms. Hydrogen atoms are drawn as spheres of arbitrary radius with hydrogen bonds drawn as dashed lines.

**Fig. 2.**
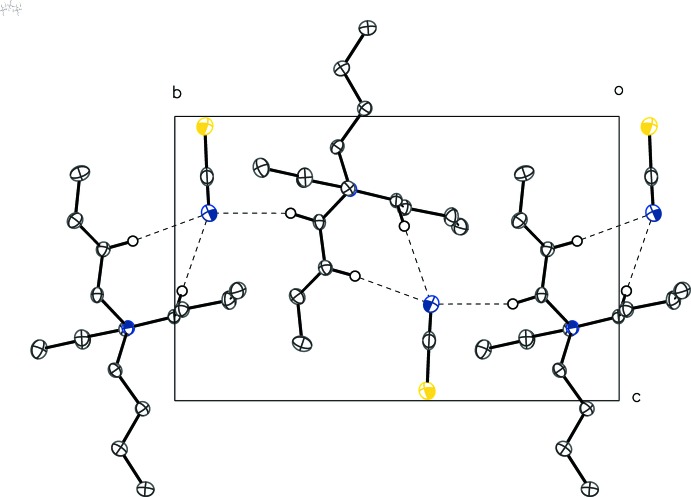
Projection along the a-axis showing the chains running along the b-axis made by the weak C—H···N interactions (dashed lines). Hydrogen atoms not involved in these interactions have been removed for clarity.

## supplementary crystallographic information

### 1. Comment

Despite the fact the title compound (I) is a common reagent used for example as a reducing agent (Hutchins & Kandasamy, 1973), its crystal structure has not yet been reported.

The structure contains distinctive N(Bu)_4_ cations and BH_3_CN anions lying in general positions (figure 1). The anion is almost linear with a N—C—B angle of 178.7 (2)°. The C—N—C angle values at the core of the tetra-*n*-butylammonium cation range from 105.74 (11)° to 111.35 (11)° with an average of 109.49 (11)° close to the ideal tetrahedral value.

The *n*-butyl chains are fully extended with an all-*trans* conformations, giving for the tetra-*n*-butylammonium cation a distorted *D*_2_*_d_* point group symmetry (Alder *et al.*, 1990).

Each anion is surrounded by two cations linked through three weak C—H···N hydrogen bonds; these define chains along the *b*-axis (figure 2) with a distance separation between the nitrogen atoms of 4.404 (2) Å and 4.491 (2) Å.

The title compound, as many tetraalkylammonium borohydride salts (Jaroń *et al.*, 2012; Jaroń & Grochala, 2011) is loosely packed with a density lower than 1.

### 2. Experimental

Compound (I) is commercially available from Sigma-Aldrich and a crystalline specimen has been extracted directly from the commercial flask.

### 3. Refinement

H-atoms were placed in calculated positions (C—H 0.98–0.99 Å, B–H 0.99 Å) and were included in the refinement in the riding model approximation, with *U*_iso_(H) = 1.2Ueq(C) for methylene or 1.5Ueq(C) for methyl groups and the hydrogen atoms linked to the boron atom.

### Crystal data

**Table:**

C_16_H_36_N^+^·CH_3_BN^−^	*F*(000) = 320
*M**_r_* = 282.31	*D*_x_ = 0.964 Mg m^−^^3^
Monoclinic, *P*2_1_	Cu *K*α radiation, λ = 1.54178 Å
*a* = 7.8312 (5) Å	Cell parameters from 9860 reflections
*b* = 13.9334 (9) Å	θ = 5.0–70.0°
*c* = 9.6313 (6) Å	µ = 0.40 mm^−^^1^
β = 112.269 (2)°	*T* = 100 K
*V* = 972.54 (11) Å^3^	Block, clear light colourless
*Z* = 2	0.25 × 0.2 × 0.15 mm

### Data collection

**Table:**

Bruker Microstar X8 diffractometer	3520 independent reflections
Radiation source: Rotating-anode X-ray tube, Bruker Microstar/FR591 generator	3510 reflections with *I* > 2σ(*I*)
Helios Mirror Optics monochromator	*R*_int_ = 0.041
Detector resolution: 8.3 pixels mm^-1^	θ_max_ = 70.2°, θ_min_ = 5.0°
ω scans	*h* = −9→8
Absorption correction: multi-scan (*SADABS*; Sheldrick, 2012)	*k* = −16→17
*T*_min_ = 0.590, *T*_max_ = 0.753	*l* = −11→11
18043 measured reflections	

### Refinement

**Table:**

Refinement on *F*^2^	Hydrogen site location: inferred from neighbouring sites
Least-squares matrix: full	H-atom parameters constrained
*R*[*F*^2^ > 2σ(*F*^2^)] = 0.035	*w* = 1/[σ^2^(*F*_o_^2^) + (0.059*P*)^2^ + 0.1174*P*] where *P* = (*F*_o_^2^ + 2*F*_c_^2^)/3
*wR*(*F*^2^) = 0.092	(Δ/σ)_max_ < 0.001
*S* = 1.03	Δρ_max_ = 0.17 e Å^−^^3^
3520 reflections	Δρ_min_ = −0.16 e Å^−^^3^
186 parameters	Absolute structure: Flack parameter determined using 1596 quotients [(I^+^)-(I^-^)]/[(I^+^)+(I^-^)] (Parsons & Flack, 2004)
1 restraint	Absolute structure parameter: 0.14 (12)
Primary atom site location: structure-invariant direct methods	

### Special details

**Table:**

Experimental. X-ray crystallographic data for I were collected from a single-crystal sample, which was mounted on a loop fiber. Data were collected using a Bruker microstar diffractometer equipped with a Platinum 135 CCD Detector, a Helios optics and a Kappa goniometer. The crystal-to-detector distance was 4.0 cm, and the data collection was carried out in 512 *x* 512 pixel mode. The initial unit-cell parameters were determined by a least-squares fit of the angular setting of strong reflections, collected by a 110.0 degree scan in 110 frames over three different parts of the reciprocal space
Geometry. All e.s.d.'s (except the e.s.d. in the dihedral angle between two l.s. planes) are estimated using the full covariance matrix. The cell e.s.d.'s are taken into account individually in the estimation of e.s.d.'s in distances, angles and torsion angles; correlations between e.s.d.'s in cell parameters are only used when they are defined by crystal symmetry. An approximate (isotropic) treatment of cell e.s.d.'s is used for estimating e.s.d.'s involving l.s. planes.
Refinement. 1. Fixed *U*_iso_ At 1.2 times of: All C(H,H) groups At 1.5 times of: All B(H,H,H) groups, All C(H,H,H) groups 2.a Secondary CH2 refined with riding coordinates: C1(H1A,H1B), C2(H2A,H2B), C3(H3A,H3B), C5(H5A,H5B), C6(H6A,H6B), C7(H7A,H7B), C9(H9A,H9B), C10(H10A,H10B), C11(H11A,H11B), C13(H13A,H13B), C14(H14A,H14B), C15(H15A,H15B) 2.b Idealized Me refined as rotating group: C8(H8A,H8B,H8C), C12(H12A,H12B,H12C), C16(H16A,H16B,H16C), C4(H4A,H4B,H4C), B1(H1C,H1D,H1E)

### Fractional atomic coordinates and isotropic or equivalent isotropic displacement parameters (Å^2^)

**Table:**

	*x*	*y*	*z*	*U*_iso_*/*U*_eq_	
N1	0.37329 (16)	0.60490 (9)	0.25684 (14)	0.0171 (3)	
C1	0.35569 (19)	0.67475 (12)	0.37167 (17)	0.0186 (3)	
H1A	0.2313	0.6701	0.3698	0.022*	
H1B	0.3712	0.7393	0.3405	0.022*	
C2	0.4898 (2)	0.66116 (12)	0.53249 (16)	0.0209 (3)	
H2A	0.6134	0.6790	0.5418	0.025*	
H2B	0.4914	0.5944	0.5616	0.025*	
C3	0.4275 (2)	0.72452 (12)	0.63373 (17)	0.0246 (4)	
H3A	0.4305	0.7911	0.6052	0.030*	
H3B	0.3008	0.7088	0.6178	0.030*	
C5	0.56503 (19)	0.61031 (11)	0.25259 (17)	0.0183 (3)	
H5A	0.5705	0.5666	0.1761	0.022*	
H5B	0.6538	0.5883	0.3483	0.022*	
C6	0.6223 (2)	0.70965 (12)	0.22094 (18)	0.0215 (3)	
H6A	0.6227	0.7536	0.2992	0.026*	
H6B	0.5336	0.7330	0.1262	0.026*	
C7	0.8133 (2)	0.70710 (12)	0.2143 (2)	0.0252 (3)	
H7A	0.9020	0.6845	0.3097	0.030*	
H7B	0.8131	0.6622	0.1373	0.030*	
C8	0.8715 (2)	0.80552 (13)	0.1804 (2)	0.0286 (4)	
H8A	0.7902	0.8255	0.0822	0.043*	
H8B	0.9956	0.8024	0.1840	0.043*	
H8C	0.8655	0.8508	0.2536	0.043*	
C9	0.2259 (2)	0.63434 (11)	0.10726 (17)	0.0193 (3)	
H9A	0.1081	0.6354	0.1187	0.023*	
H9B	0.2520	0.6995	0.0857	0.023*	
C10	0.2053 (2)	0.57252 (12)	−0.02823 (17)	0.0224 (3)	
H10A	0.1533	0.5106	−0.0197	0.027*	
H10B	0.3249	0.5620	−0.0338	0.027*	
C11	0.0779 (2)	0.62444 (14)	−0.16886 (18)	0.0274 (4)	
H11A	−0.0376	0.6389	−0.1581	0.033*	
H11B	0.1343	0.6848	−0.1782	0.033*	
C12	0.0379 (3)	0.56601 (14)	−0.31121 (18)	0.0319 (4)	
H12A	−0.0299	0.6046	−0.3970	0.048*	
H12B	−0.0337	0.5105	−0.3090	0.048*	
H12C	0.1520	0.5460	−0.3175	0.048*	
C13	0.3451 (2)	0.50172 (11)	0.29578 (16)	0.0187 (3)	
H13A	0.3467	0.4603	0.2153	0.022*	
H13B	0.4484	0.4835	0.3859	0.022*	
C14	0.1676 (2)	0.48347 (12)	0.32089 (18)	0.0222 (3)	
H14A	0.0628	0.5025	0.2323	0.027*	
H14B	0.1668	0.5219	0.4046	0.027*	
C15	0.1506 (2)	0.37789 (13)	0.3535 (2)	0.0276 (4)	
H15A	0.2592	0.3582	0.4385	0.033*	
H15B	0.1452	0.3399	0.2675	0.033*	
C16	−0.0207 (3)	0.35851 (13)	0.3874 (2)	0.0310 (4)	
H16A	−0.1288	0.3712	0.2996	0.047*	
H16B	−0.0202	0.3995	0.4677	0.047*	
H16C	−0.0211	0.2926	0.4165	0.047*	
C4	0.5460 (2)	0.71352 (15)	0.79966 (19)	0.0313 (4)	
H4A	0.6699	0.7334	0.8177	0.047*	
H4B	0.5462	0.6475	0.8284	0.047*	
H4C	0.4965	0.7527	0.8576	0.047*	
N2	0.5861 (3)	0.42280 (13)	0.66014 (18)	0.0418 (4)	
C17	0.6380 (2)	0.42768 (13)	0.7884 (2)	0.0297 (4)	
B1	0.7120 (3)	0.43232 (17)	0.9652 (2)	0.0332 (4)	
H1C	0.6112	0.4235	0.9975	0.050*	
H1D	0.7681	0.4937	0.9987	0.050*	
H1E	0.8018	0.3826	1.0069	0.050*	

### Atomic displacement parameters (Å^2^)

**Table:**

	*U*^11^	*U*^22^	*U*^33^	*U*^12^	*U*^13^	*U*^23^
N1	0.0163 (6)	0.0160 (7)	0.0194 (6)	0.0013 (4)	0.0071 (5)	0.0006 (5)
C1	0.0192 (7)	0.0154 (7)	0.0236 (7)	0.0018 (5)	0.0108 (6)	−0.0010 (6)
C2	0.0220 (7)	0.0194 (8)	0.0221 (7)	0.0006 (6)	0.0092 (6)	−0.0012 (6)
C3	0.0262 (7)	0.0248 (9)	0.0251 (8)	0.0011 (6)	0.0122 (6)	−0.0034 (7)
C5	0.0140 (6)	0.0199 (8)	0.0214 (7)	0.0022 (5)	0.0072 (6)	−0.0007 (6)
C6	0.0198 (7)	0.0207 (8)	0.0265 (8)	0.0002 (6)	0.0117 (6)	0.0003 (6)
C7	0.0196 (7)	0.0232 (8)	0.0351 (8)	0.0000 (6)	0.0130 (6)	−0.0007 (7)
C8	0.0257 (8)	0.0270 (9)	0.0367 (9)	−0.0059 (6)	0.0158 (7)	−0.0028 (7)
C9	0.0176 (7)	0.0191 (7)	0.0206 (7)	0.0026 (6)	0.0065 (6)	0.0025 (6)
C10	0.0239 (7)	0.0213 (8)	0.0208 (7)	0.0024 (6)	0.0070 (6)	0.0007 (6)
C11	0.0310 (8)	0.0250 (9)	0.0226 (8)	0.0028 (7)	0.0062 (7)	0.0027 (6)
C12	0.0387 (9)	0.0298 (10)	0.0215 (8)	0.0001 (8)	0.0049 (7)	0.0009 (7)
C13	0.0203 (7)	0.0143 (7)	0.0203 (7)	0.0001 (6)	0.0064 (5)	0.0005 (6)
C14	0.0235 (7)	0.0198 (8)	0.0240 (7)	−0.0014 (6)	0.0099 (6)	0.0005 (6)
C15	0.0274 (8)	0.0209 (8)	0.0336 (8)	−0.0053 (7)	0.0104 (7)	0.0017 (7)
C16	0.0374 (9)	0.0296 (10)	0.0282 (8)	−0.0132 (7)	0.0148 (7)	−0.0027 (7)
C4	0.0336 (8)	0.0383 (10)	0.0243 (8)	−0.0005 (8)	0.0134 (7)	−0.0056 (7)
N2	0.0627 (11)	0.0240 (8)	0.0286 (8)	−0.0016 (8)	0.0059 (7)	0.0025 (7)
C17	0.0335 (8)	0.0164 (8)	0.0341 (9)	0.0002 (7)	0.0070 (7)	0.0010 (7)
B1	0.0389 (10)	0.0263 (10)	0.0306 (10)	0.0033 (9)	0.0088 (8)	−0.0021 (8)

### Geometric parameters (Å, º)

**Table:**

N1—C1	1.5182 (18)	C10—H10B	0.9700
N1—C5	1.5195 (17)	C10—C11	1.526 (2)
N1—C9	1.5216 (18)	C11—H11A	0.9700
N1—C13	1.5229 (19)	C11—H11B	0.9700
C1—H1A	0.9700	C11—C12	1.521 (2)
C1—H1B	0.9700	C12—H12A	0.9600
C1—C2	1.518 (2)	C12—H12B	0.9600
C2—H2A	0.9700	C12—H12C	0.9600
C2—H2B	0.9700	C13—H13A	0.9700
C2—C3	1.526 (2)	C13—H13B	0.9700
C3—H3A	0.9700	C13—C14	1.5192 (19)
C3—H3B	0.9700	C14—H14A	0.9700
C3—C4	1.521 (2)	C14—H14B	0.9700
C5—H5A	0.9700	C14—C15	1.520 (2)
C5—H5B	0.9700	C15—H15A	0.9700
C5—C6	1.521 (2)	C15—H15B	0.9700
C6—H6A	0.9700	C15—C16	1.521 (2)
C6—H6B	0.9700	C16—H16A	0.9600
C6—C7	1.5215 (19)	C16—H16B	0.9600
C7—H7A	0.9700	C16—H16C	0.9600
C7—H7B	0.9700	C4—H4A	0.9600
C7—C8	1.519 (2)	C4—H4B	0.9600
C8—H8A	0.9600	C4—H4C	0.9600
C8—H8B	0.9600	N2—C17	1.147 (2)
C8—H8C	0.9600	C17—B1	1.578 (3)
C9—H9A	0.9700	B1—H1C	0.9600
C9—H9B	0.9700	B1—H1D	0.9600
C9—C10	1.520 (2)	B1—H1E	0.9600
C10—H10A	0.9700		
			
C1—N1—C5	110.57 (11)	C9—C10—H10B	110.0
C1—N1—C9	105.74 (11)	C9—C10—C11	108.33 (13)
C1—N1—C13	111.35 (11)	H10A—C10—H10B	108.4
C5—N1—C9	111.33 (10)	C11—C10—H10A	110.0
C5—N1—C13	106.90 (10)	C11—C10—H10B	110.0
C9—N1—C13	111.03 (11)	C10—C11—H11A	109.0
N1—C1—H1A	108.2	C10—C11—H11B	109.0
N1—C1—H1B	108.2	H11A—C11—H11B	107.8
N1—C1—C2	116.39 (12)	C12—C11—C10	112.85 (15)
H1A—C1—H1B	107.3	C12—C11—H11A	109.0
C2—C1—H1A	108.2	C12—C11—H11B	109.0
C2—C1—H1B	108.2	C11—C12—H12A	109.5
C1—C2—H2A	110.0	C11—C12—H12B	109.5
C1—C2—H2B	110.0	C11—C12—H12C	109.5
C1—C2—C3	108.34 (12)	H12A—C12—H12B	109.5
H2A—C2—H2B	108.4	H12A—C12—H12C	109.5
C3—C2—H2A	110.0	H12B—C12—H12C	109.5
C3—C2—H2B	110.0	N1—C13—H13A	108.5
C2—C3—H3A	108.9	N1—C13—H13B	108.5
C2—C3—H3B	108.9	H13A—C13—H13B	107.5
H3A—C3—H3B	107.7	C14—C13—N1	115.09 (12)
C4—C3—C2	113.43 (13)	C14—C13—H13A	108.5
C4—C3—H3A	108.9	C14—C13—H13B	108.5
C4—C3—H3B	108.9	C13—C14—H14A	109.5
N1—C5—H5A	108.6	C13—C14—H14B	109.5
N1—C5—H5B	108.6	C13—C14—C15	110.59 (13)
N1—C5—C6	114.83 (12)	H14A—C14—H14B	108.1
H5A—C5—H5B	107.5	C15—C14—H14A	109.5
C6—C5—H5A	108.6	C15—C14—H14B	109.5
C6—C5—H5B	108.6	C14—C15—H15A	109.3
C5—C6—H6A	109.5	C14—C15—H15B	109.3
C5—C6—H6B	109.5	C14—C15—C16	111.69 (15)
C5—C6—C7	110.87 (12)	H15A—C15—H15B	107.9
H6A—C6—H6B	108.1	C16—C15—H15A	109.3
C7—C6—H6A	109.5	C16—C15—H15B	109.3
C7—C6—H6B	109.5	C15—C16—H16A	109.5
C6—C7—H7A	109.3	C15—C16—H16B	109.5
C6—C7—H7B	109.3	C15—C16—H16C	109.5
H7A—C7—H7B	108.0	H16A—C16—H16B	109.5
C8—C7—C6	111.58 (13)	H16A—C16—H16C	109.5
C8—C7—H7A	109.3	H16B—C16—H16C	109.5
C8—C7—H7B	109.3	C3—C4—H4A	109.5
C7—C8—H8A	109.5	C3—C4—H4B	109.5
C7—C8—H8B	109.5	C3—C4—H4C	109.5
C7—C8—H8C	109.5	H4A—C4—H4B	109.5
H8A—C8—H8B	109.5	H4A—C4—H4C	109.5
H8A—C8—H8C	109.5	H4B—C4—H4C	109.5
H8B—C8—H8C	109.5	N2—C17—B1	178.7 (2)
N1—C9—H9A	108.0	C17—B1—H1C	109.5
N1—C9—H9B	108.0	C17—B1—H1D	109.5
H9A—C9—H9B	107.3	C17—B1—H1E	109.5
C10—C9—N1	117.12 (12)	H1C—B1—H1D	109.5
C10—C9—H9A	108.0	H1C—B1—H1E	109.5
C10—C9—H9B	108.0	H1D—B1—H1E	109.5
C9—C10—H10A	110.0		
			
N1—C1—C2—C3	−169.39 (12)	C5—N1—C13—C14	174.17 (12)
N1—C5—C6—C7	−178.31 (12)	C5—C6—C7—C8	179.12 (13)
N1—C9—C10—C11	−169.54 (12)	C9—N1—C1—C2	−179.68 (12)
N1—C13—C14—C15	178.00 (12)	C9—N1—C5—C6	60.80 (16)
C1—N1—C5—C6	−56.43 (15)	C9—N1—C13—C14	−64.23 (15)
C1—N1—C9—C10	−176.84 (12)	C9—C10—C11—C12	−176.50 (14)
C1—N1—C13—C14	53.31 (15)	C13—N1—C1—C2	59.62 (15)
C1—C2—C3—C4	177.11 (14)	C13—N1—C5—C6	−177.78 (12)
C5—N1—C1—C2	−59.06 (16)	C13—N1—C9—C10	−55.94 (16)
C5—N1—C9—C10	63.04 (16)	C13—C14—C15—C16	176.90 (13)

### Hydrogen-bond geometry (Å, º)

**Table:**

*D*—H···*A*	*D*—H	H···*A*	*D*···*A*	*D*—H···*A*
C1—H1*B*···N2^i^	0.97	2.58	3.515 (2)	162
C2—H2*B*···N2	0.97	2.58	3.523 (2)	165
C13—H13*B*···N2	0.97	2.59	3.474 (2)	152

Symmetry code: (i) −*x*+1, *y*+1/2, −*z*+1.
